# RETRACTION: Resveratrol Downregulates Interleukin‐6‐Stimulated Sonic Hedgehog Signaling in Human Acute Myeloid Leukemia

**DOI:** 10.1155/ecam/9796061

**Published:** 2026-02-25

**Authors:** Evidence-Based Complementary and Alternative Medicine

RETRACTION: Y.‐C. Su, S.‐C. Li, Y.‐C. Wu, L.‐M. Wang, K. S. Clifford Chao, and H.‐F. Liao, “Resveratrol Downregulates Interleukin‐6‐Stimulated Sonic Hedgehog Signaling in Human Acute Myeloid Leukemia,” *Evidence-Based Complementary and Alternative Medicine*, vol. 2013 (2013), https://doi.org/10.1155/2013/547430.

The above article, published online on 20 February 2013 in Wiley Online Library (https://wileyonlinelibrary.com), has been retracted by John Wiley & Sons Ltd.

The retraction has been agreed following an investigation of the issues raised by *Actinopolyspora biskrensis* on PubPeer [1], which highlighted concerns related to multiple figures in the manuscript.

More specifically, the Western Blot bands corresponding to the expression of C‐Shh and N‐Shh in Figure 3b are unexpectedly similar after a horizontal stretch of the latter. Further concerns have been identified regarding overlapping features of the “Resveratrol,” “IL‐6+Resveratrol,” and “IL‐6+cyclopamine” panels in Figure 5b.

The authors were contacted to elaborate on these concerns and provide the raw data supporting the conclusions of the article; however, they were unable to do so. As a result of this investigation, the results of the article are no longer considered reliable.

The authors disagree with this retraction.
